# A highly invasive subpopulation of MDA-MB-231 breast cancer cells shows accelerated growth, differential chemoresistance, features of apocrine tumors and reduced tumorigenicity *in vivo*

**DOI:** 10.18632/oncotarget.11931

**Published:** 2016-09-10

**Authors:** Adriana Amaro, Giovanna Angelini, Valentina Mirisola, Alessia Isabella Esposito, Daniele Reverberi, Serena Matis, Massimo Maffei, Walter Giaretti, Maurizio Viale, Rosaria Gangemi, Laura Emionite, Simonetta Astigiano, Michele Cilli, Beatrice E. Bachmeier, Peter H. Killian, Adriana Albini, Ulrich Pfeffer

**Affiliations:** ^1^ Molecular Pathology, IRCCS AOU San Martino – IST Istituto Nazionale per la Ricerca sul Cancro, Genova, Italy; ^2^ Biotherapy, IRCCS AOU San Martino – IST Istituto Nazionale per la Ricerca sul Cancro, Genova, Italy; ^3^ Animal Facility, IRCCS AOU San Martino – IST Istituto Nazionale per la Ricerca sul Cancro, Genova, Italy; ^4^ Immunology, IRCCS AOU San Martino – IST Istituto Nazionale per la Ricerca sul Cancro, Genova, Italy; ^5^ Institute of Laboratory Medicine, Ludwig-Maximilians-University, Munich, Germany; ^6^ Scientific and Technology Park, IRCCS MultiMedica, Milan, Italy

**Keywords:** breast cancer, invasion, apocrine breast cancer, metastasis, aneuploidy

## Abstract

The acquisition of an invasive phenotype is a prerequisite for metastasization, yet it is not clear whether or to which extent the invasive phenotype is linked to other features characteristic of metastatic cells. We selected an invasive subpopulation from the triple negative breast cancer cell line MDA-MB-231, performing repeated cycles of preparative assays of invasion through *Matrigel* covered membranes. The invasive sub-population of MDA-MB-231 cells exhibits stronger migratory capacity as compared to parental cells confirming the highly invasive potential of the selected cell line. Prolonged cultivation of these cells did not abolish the invasive phenotype. ArrayCGH, DNA index quantification and karyotype analyses confirmed a common genetic origin of the parental and invasive subpopulations and revealed discrete structural differences of the invasive subpopulation including increased ploidy and the absence of a characteristic amplification of chromosome 5p14.1-15.33. Gene expression analyses showed a drastically altered expression profile including features of apocrine breast cancers and of invasion related matrix-metalloproteases and cytokines. The invasive cells showed accelerated proliferation, increased apoptosis, and an altered pattern of chemo-sensitivity with lower IC50 values for drugs affecting the mitotic apparatus. However, the invasive cell population is significantly less tumorigenic in orthotopic mouse xenografts suggesting that the acquisition of the invasive capacity and the achievement of metastatic growth potential are distinct events.

## INTRODUCTION

Breast cancer is the most frequent form of cancer among women and still represents a major cause of death in women due to cancer. Breast cancer develops from atypical hyperplasia, a premalignant lesion that, via ductal carcinoma in situ (DCIS) progresses to invasive carcinoma with varying metastatic potential. This process is believed to be driven by successive mutations as predicted by the multistep carcinogenesis model [1, 2]. Following this model distinct gene expression profiles are expected to be associated with the different stages of cancer development. However, a multitude of gene expression studies performed to identify cancer progression related genes did not succeed in identifying an unequivocal progression signature [3-9]. In addition, one of the first gene expression studies, performed by Ma and colleagues, revealed the presence of only slight variations in gene expression profiles among the distinct pathological steps within the same patients [4]. It is therefore not clear which and how many genes are involved in the transformation of a hyperplasia into carcinoma and which genes drive the transition from DCIS to an invasive carcinoma. Despite the fact that gene expression classifiers of primary breast cancers can predict the metastatic risk with a certain accuracy [10] it is still matter of discussion whether or to which extent the metastatic phenotype is predetermined by the driver mutations present in early stages or acquired by additional mutations later on during cancer development [11-15].

Metastasization consists in several distinct steps: invasion of the surrounding tissue, entry into the blood stream, survival in the absence of anchorage (anti-anoikis), tethering to the vascular wall of vessels in metastasis target tissues, induction of necroptosis of endothelial cells, extravasation, colonization of the target tissue, growth in response to local growth factors and immune escape [16-19]. Each of these steps requires specific molecular events in terms of gene and protein expression and eventually, but not necessarily, somatic mutations. These molecular events are far from being completely described. The different steps are expected to be to a certain degree independent of each other and each transition determines a selection of cells that have acquired the molecular alterations needed to perform the following task. This makes cancer metastasis a highly inefficient process. From the hundreds of thousands or millions of cells released from primary cancers each day [20, 21], only a very tiny subpopulation will make it through this “decathlon” [22], while the vast majority of cells die within a few days or re-seed the same tumor site [23]. Hence, each single step is a target for prevention of metastasis with the aim to reduce the probability of the cancer cell to perform the next step [17].

Since cancer development cannot be studied as it occurs we must rely on cellular models for the identification of molecular players. The invasive phenotype is characterized by the potential of cells of the primary cancer to degrade the extracellular matrix and to invade the surrounding tissue [24, 25]. In order to obtain a more detailed picture of the molecular events typical of the invasive tumor cell, we selected and thoroughly characterized a highly invasive subpopulation from the triple negative breast cancer cell line MDA-MB-231 that is known to be able to give lung [26, 27], bone [28, 29] or brain [30] specific metastases in mouse models. The target site to which MDA-MB-231 cells metastasize depends on specific gene expression alterations that are most likely important in the later steps of metastasization consisting in extravasation and growth in the target tissue [27, 29, 30]. Here we focus on the first step of this process, on invasion, that is most likely independent of the final target tissue of metastasis. The phenotypic, genetic, molecular and functional characterization of the invasive subpopulation obtained reveals that the invasive phenotype is independent of the tumorigenic potential and of drug sensitivity.

## RESULTS

### Isolation of an invasive subpopulation within MDA-MB-231 cell lines

**Figure 1:**
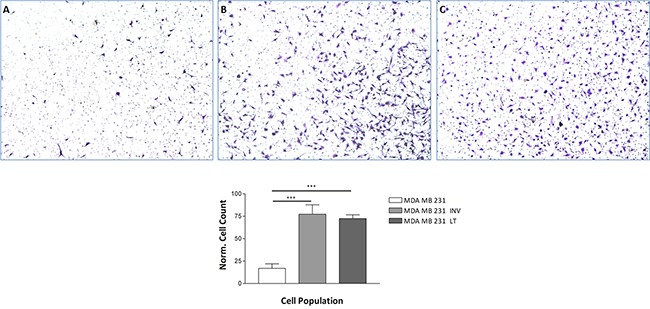
Invasion through Matrigel The invasive potential of MDA-MB-231 cells, **231, A.** the invasive subpopulation, MDA-MB-INV cells, selected therefrom after three cycles of selection INV, **B.** and the selected cells after continuous growth for six months, MDA-MB-LT cells LT, **C.** was analyzed in Matrigel covered Transwell chambers. Invaded cells were counted. INV and LT cells show a significantly increased invasion potential as compared to 231 cells. The numbers of invaded cells counted was normalized for proliferation at 24 hrs. using the proliferation assay shown in Figure 2.

We isolated an invasive subpopulation from the triple negative breast cancer cell line MDA-MB-231 using repeated cycles of preparative assays of invasion, through *Matrigel* covered membranes. The number of MDA-MB-231 Invasive cells that passed through the *Matrigel* membrane in 24 hours was approximately 6 times higher than the parental 231 (Figure 1A and 1B). The invasive phenotype of the selected subpopulation is stable upon prolonged cultivation since the cells still showed increased invasivity in the Matrigel assay (Figure 1C). We will refer to these populations as 231 (parental cell line), INV (selected invasive subpopulation) and LT (invasive subpopulation after long term cultivation) in the following. INV cells have been obtained from 231 cells by preparative invasion assays and LT cells have been obtained from INV cells through continuous cultivation for six months with biweekly splitting.

Enhanced proliferation (see below) could lead to an apparently increased invasion although the cells are kept in medium with low serum levels for the invasion assay. Yet once invaded, the more INV and LT cells could proliferate more rapidly. We therefore normalized the invasion data for proliferation.

### Phenotypic and functional characterization of the invasive subpopulations

**Figure 2:**
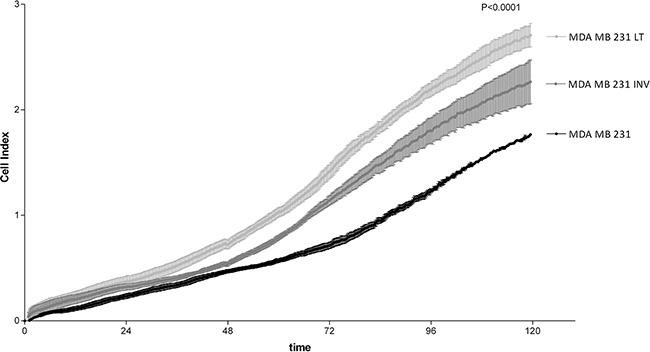
Analysis of cell growth 231, INV and LT cells were analyzed for cell growth by real time electrical impedance measurements (xCELLigence System). INV and LT grow significantly more rapidly than 231 cells. The difference between LT and INV cells is also highly significant (p <0.0001 for all comparisons).

Cell growth of the three populations was assessed by the colorimetric test crystal violet proliferation assay (data not shown) and, in parallel, by the *xCELLigence System* that allowed continuous monitoring of cell growth over 5 days (Figure 2). The results using both approaches were overlapping and, as shown in Figure 2, the invasive phenotype (INV and LT cells) displayed a statistically significantly increased cell growth as compared to the WT cells. LT cells showed a significantly elevated cell growth even when compared with INV cells (Figure 2).

**Figure 3:**
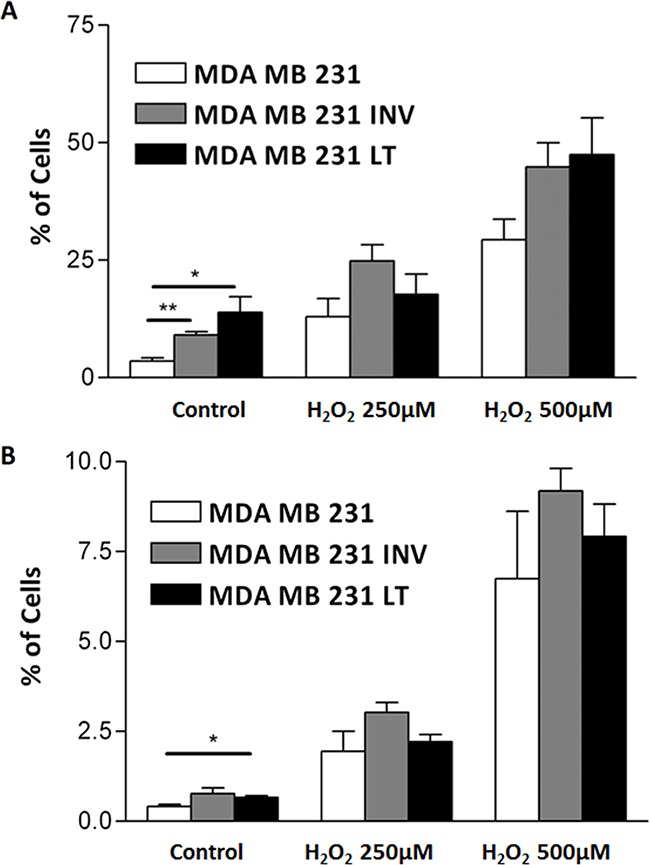
Analysis of spontaneous and induced apoptosis Spontaneous (Control) and with 250μM or 500μM H_2_O_2_ induced apoptosis **A.** and necrosis **B.** were measured by flow cytometry after Annexin-V-FLUOS staining of 231, INV and LT cells. INV and LT cells show significantly increased spontaneous apoptosis as compared to 231 cells. The response to superoxide also showed a slight increase that did not reach significance.

In order to establish whether increased cell growth was due to increased proliferation or reduced apoptosis we evaluated, by flow cytometry, apoptosis and necrosis rates of the 231 cells and the two subpopulations under standard growth conditions or after H_2_O_2_ treatment. H_2_O_2_ induced apoptosis and necrosis in all three populations. INV and LT cells appear more prone to undergo apoptosis (Figure 3a) and necrosis (Figure 3b), this reaches significance only for the growth in the absence of the apoptotic stimulus.

We then performed structural genomic analyses in order to control the genetic relation of the three populations and to identify eventual genomic alterations. Hybridization of genomic DNA to SNP arrays revealed a SNP call concordance of 96.87% and 96.91% for INV and LT versus 231 cells, respectively, clearly indicating a genetic relation among the populations and excluding cell contamination.

**Figure 4:**
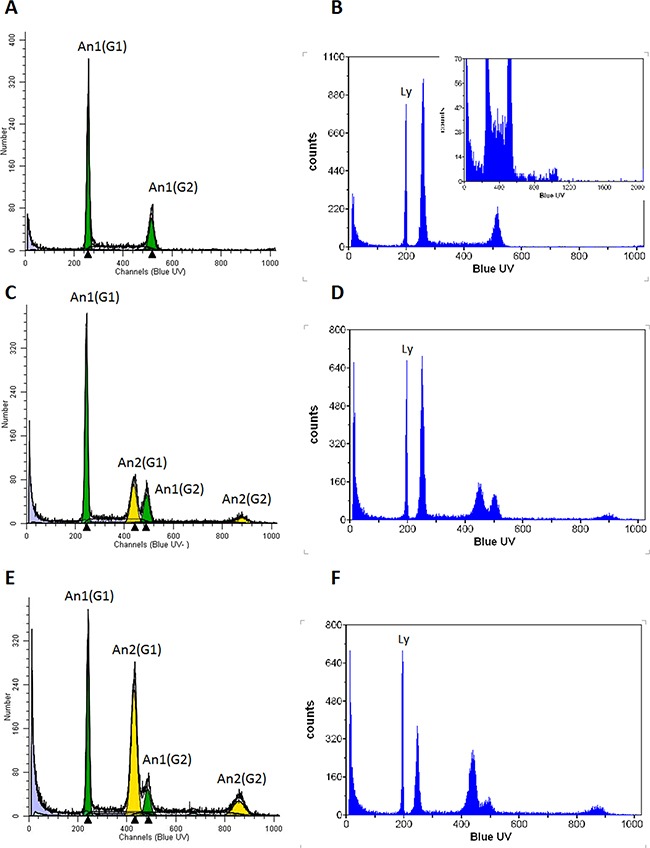
Flow cytometric determination of DNA content 231 **A, B.** INV **C, D.** and LT **E, F.** cells were analyzed after DAPI staining for their DNA content. The G1 peak of 231 cells shows 1.3 times the content of normal human lymphocytes (Ly; indicated in panels B,D,F), compatible with reports of the chromosome content of these cells (aneuploid clone 1, AN1). The inclusion of channels over 1000 for cells with higher DNA content (insert in B) shows the presence of a small population with higher DNA content approximately twice that of the G2/M phase of AN1 (aneuploidy clone 2, AN2). The AN1 population is shown in green, the AN2 population in yellow. Both populations show their G1 and G2/M peaks. The panels B,D and F show the same analysis with the addition of human female lymphocytes with the normal diploid human genome for comparison.

Analysis of the DNA content by flow cytometry revealed the presence of two subpopulations in the INV population (Figure 4c and 4d), one with a DNA index indistinguishable from the parental 231 cells (Figure 4a and 4b) that we referred to as AN1 and another with an almost doubled DNA content (AN2). The AN2 peak is clearly distinct from the G2/M peak of 231 cells and INV cells also show a peak with a DNA content twice of that of the AN2 peak corresponding to AN2 G2/M cells (Figure 4c). Since the INV cells have been obtained from the 231 population, we expected the AN2 population to be present, though at a lower ratio, also in the 231 population. The G1 AN2 peak cannot be detected since it is too close to the G2/M AN1 peak but the G2/M AN2 peak is detectable when zooming to regions of higher DNA content (channels over 1000, insert to Figure 4b). Hence, the population that has been selected by the preparative invasion assays is also present in the parental 231 cell line. Table 1 reports the DNA indices measured and the corresponding percentage of the population. The AN2 peaks correspond to a subpopulation of cells with almost the double DNA content as compared to 231 cells (DNA Index 1.30 and 2.28 for 231 and AN2-INV cells, respectively). LT cells show the same two populations with, however, an enrichment of the AN2 peaks (59.52%, Figure 4e and 4f) consistent with the observed increased growth rate that is expected to determine an enrichment of the faster growing subpopulation. The slight differences in the DNA indices measured for the AN2 peak in INV and LT populations are beyond the resolution limit of this analysis.

**Table 1:** DNA content of MDA-MB-231 subpopulations

DNA content of MDA-MB-231 subpopulations				
		DNA INDEX	CV (%)	Fraction (%)
AN1	231	1.3	2.29	100
	INV	1.28	2.38	68.31
	LT	1.26	2.55	40.48
AN2	231	nd	nd	nd
	INV	2.28	3.67	31.69
	LT	2.24	3.63	59.52

nd = not detected using standard settings

**Figure 5:**
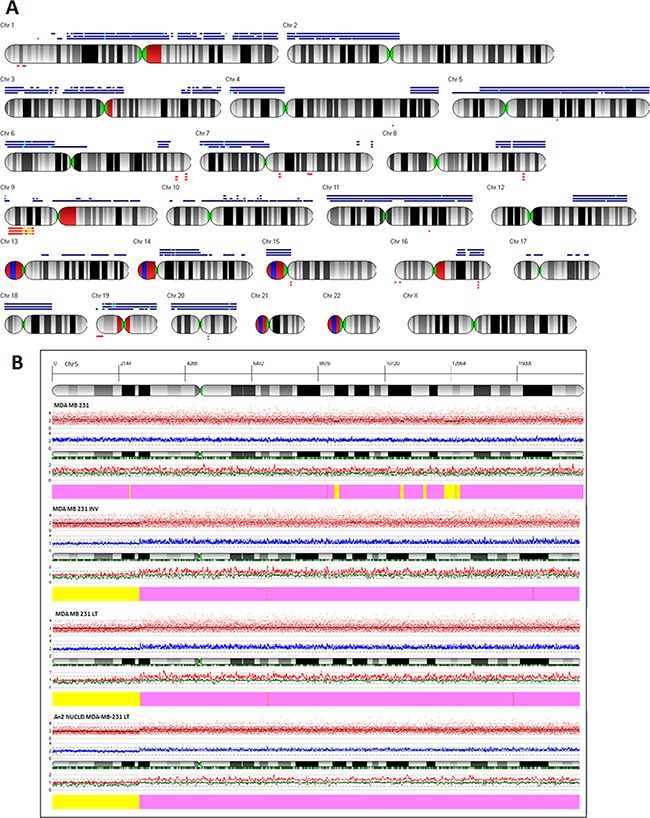
Virtual karyotyping **A.** The virtual karyotype obtained through SNP-array analysis is shown for LT, INV and 231 cells. Blue bars above the chromosome scheme indicate regions of copy number gain, red bars below them indicate copy number loss. Bars are from top to bottom: LT, INV, 231 cells. In addition to several minor differences a major difference between INV and LT cells as compared to 231 cells is observed for chromosome 5p where 231 cells show a copy number gain that is not observed for the other two populations. **B.** ArrayCGH diagram of chromosome 5 for (from top to bottom) 231, INV, and LT cells as well as for DNA obtained from nuclei of LT cells after flow cytometric isolation of the AN2 population. The whole chromosome 5 except for the distal half of the short arm 5p shows a copy number gain in all cells analyzed. Only 231 cells show a gain also for 5p. The diagram shows (from top to bottom) the coordinates of chromosome 5, the banding pattern, the actual copy number values for each single probeset (red dots), the deduced copy number applying a 10 SNP window (blue line), the position of the single probesets on the chromosome (vertical bars), the copy numbers of the two alleles (red and black lines), the interpretation (thick bar at the bottom, yellow = copy number 2N, pink = copy number gain).

In order to further analyze structural genomic differences between the parental and the invasive subpopulations we performed SNP array analyses. Figure 5a shows the virtual karyotypes of 231, INV and LT cells (see extended karyotypes in Supplementary Figure S1). Extended regions of copy number gain detected for chromosomes 9, 10, 13, 17 in 231 are absent from INV and LT cells. INV and LT cells might have lost extra copies of these chromosomes while duplicating the whole set of chromosomes. Chromosome 5 shows a general copy number gain for 231 cells that is visible also for INV and LT cells with the exception of a well-defined region of chr:5 from 5p14.1 to the telomere (Figure 5b). In addition, many copy number alterations of shorter extensions are visible (Figure 5). Since all the subpopulations are composed of AN1 and AN2 cells with the latter being abundant only in INV and LT subpopulations, not all CNA events reach sufficient consistency to be called by the algorithm used for copy number calculation. We therefore obtained a pure AN2 population by flow cytometry sorting of nuclei isolated from LT cells and analyzed the DNA isolated by aCGH (MDA-MB-231-“tetraploid”). As shown in Figure 5b, the copy number pattern of the AN2 component is similar to the pattern of the INV and LT, including the lack of amplification of chr:5p14.1-15.33. The list of genes contained in this region is reported in Supplementary Table S1.

### Chemo-sensitivity analysis

The further functional characterization was conducted with the aim to verify whether the LT population shows a more aggressive phenotype in addition to the increased invasive and proliferative potential described above. We therefore asked whether the 231 and LT cells show different sensitivities to chemotherapeutic drugs of the main classes of classical anticancer drugs most commonly used for the treatment of breast cancer. 5-fluorouracil is an antimetabolite; ifosfamide is an alkylating agent, vincristine and taxol are anti-microtubule agents with different inhibiting effects on microtubule function, irinotecan, doxorubicin and mitoxantrone are topoisomerase inhibitors, and cisplatin is an alkylating-like Pt-containing drug.

**Figure 6:**
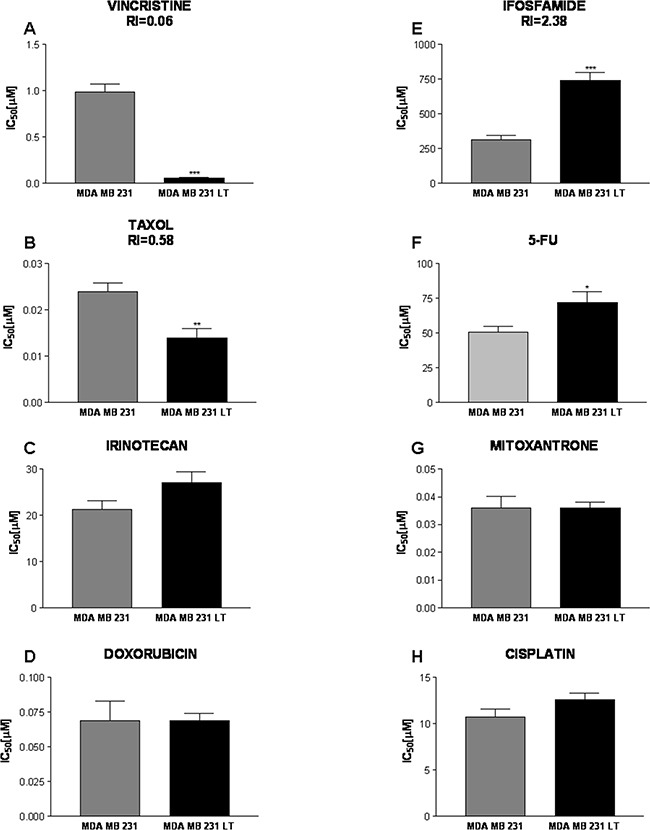
Response to chemotherapeutic drugs The effects of Vincristine **A.** Taxol **B.** Irinotecan **C.** Doxorubicin **D.** Ifosfamide **E.** 5-Fluorouracile **F.** Mitoxantrone **G.** and Cisplatin **H.** were assessed for 231 and LT cells. IC_50_ [μM] values are shown. LT cells showed a significantly increased sensitivity to Vincristine and Irinotecan (response index 0.06 and 0.58, respectively) and were more resistant to Ifosfamide (RI=2.38). LT cells also show a slightly reduced sensitivity to 5-Fluorouracile as compared to 231 cells. No significant differences were observed for the other drugs.

LT cells show significantly lower IC_50_ values for Vincristine than parental cells (RI = 0.06; Figure 6a). Vincristine binds to tubulin dimers, inhibiting assembly of microtubule structures. Disruption of the microtubules arrests mitosis in metaphase. Enhanced sensitivity is also observed for Taxol (Paclitaxel, RI = 0.58; Figure 6b). Paclitaxel also acts on the mitotic apparatus where it interferes with the breakdown of microtubules during cell division. On the contrary, LT cells show reduced sensitivity (increased IC_50_ values) to the alkylating agent Ifosfamide (Figure 6e) and to the nucleotide analog 5-fluorouracil (RI=2.4 and 1.4, respectively). For both drugs, however, the threshold of 2.5 arbitrarily chosen to define a pharmacologically significant change in sensitivity is not reached. No significant differences were observed for the other drugs (Figure 6c, 6d, 6f-6h).

### Gene expression analysis

**Figure 7:**
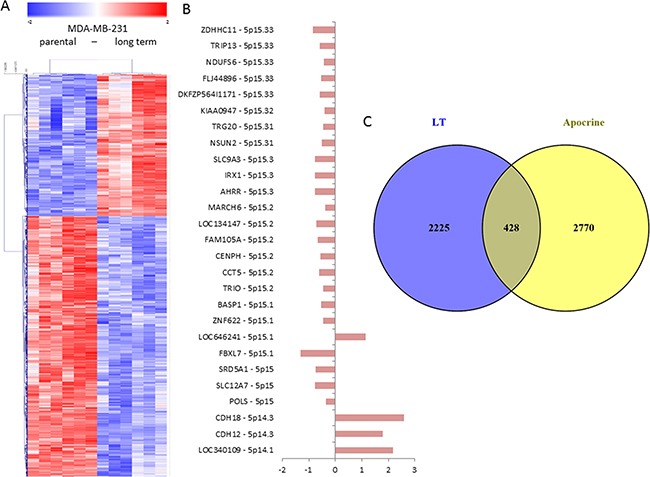
Gene expression profiling of 231 and LT cells **A.** Microarray gene expression profiles of 231 and LT cells were analyzed using SAM statistics. The expression values for significantly differentially expressed genes were clustered using hierarchical clustering (Pearson correlation distance measure, average linkage). Each column represents a single replicate of 231 or LT cells, each row represents a single probeset. Genes with expression values over the mean are shown in red, those with values below the mean in blue, mean = white. Clearly distinct expression patterns are observed for the two cell populations. **B.** The analysis of the genes from chromosome 5p among the significantly differentially expressed genes shows that most of them show reduced expression (left side of the axis) in LT cells as compared to 231 cells in concordance with the observed copy number gain in 231 cells. **C.** The analysis of bona fide targets of the inflammatory transcription factor NFkB among the genes that are significantly differentially expressed in LT cells shows that 12 of them are upregulated and 11 are down-regulated in LT cells as compared to 231 cells. **D.** The comparison of the genes differentially expressed in 231 cells with genes differentially expressed in apocrine breast cancers as compared to breast cancer of different subtypes [32] shows 428 genes that are common in both gene lists.

We expected that the profound differences in viability and drug response of the highly invasive LT population as compared to the parental cells would be reflected on the level of gene expression, thus we performed microarray gene expression analyses. The gene expression profile of LT cells was compared to 231 cells preparing six biological replicates for each population. Rigorous statistical testing applying the bootstrapping algorithm Significance Analysis of Microarray setting the false discovery rate to 0% yielded 934 and 1719 probesets that were significantly up- and downregulated, respectively, in LT cells as compared to 231 cells (Supplementary Table S2). These probesets were used for the hierarchical clustering analysis shown in Figure 7a. The strong difference in gene expression between the two populations and relatively low variability between the biological replicates of each population determine clearly distinct clusters indicating profound phenotypic differences between the two populations consistent with the genomic alterations and the functional differences observed. Among the differentially expressed genes we also observed genes located on the tip of chromosome 5p that shows amplification in 231 but not in LT cells (Figure 7b) indicating functional consequences of this copy number alteration. As expected, most of these genes are expressed at lower levels in the LT cells that also show a lower copy number.

In order to obtain a general view on the functional categories of the genes that are differentially expressed in LT versus 231 cells, we performed an enrichment analysis using EnrichR, the most exhaustive gene list comparison tool available [31]. When comparing our gene list with published gene lists, the most significant hit (adjusted p-value = 8 x 10^−9^) was a list of genes characteristically expressed in apocrine breast cancer [32]. 428 genes that are differentially expressed in LT versus 231 cells are associated with apocrine breast cancer (Figure 7c). However, expression of the androgen receptor gene (AR), that is characteristic of apocrine breast cancer, is unaltered in LT cells. MDA-MB-231 cells also express other markers of apocrine breast cancer [32] such as prolactin-induced protein, PIP, epidermal growth factor receptor, EGFR, and, at particularly high levels, 3-hydroxy-3-methylglutaryl-Coenzyme A reductase, HMGCR, but not growth hormone receptor, GHR, and prolactin receptor, PRLR. LT cells therefore share some but not all features of apocrine breast cancer. Among the genes upregulated in apocrine breast cancer and LT cells there were several cytokines and the MMPs, indicating that induction of NFkB might explain at least in part this similarity. Inflammation has been reported to be among the characteristics of apocrine breast cancer [33].

**Figure 8:**
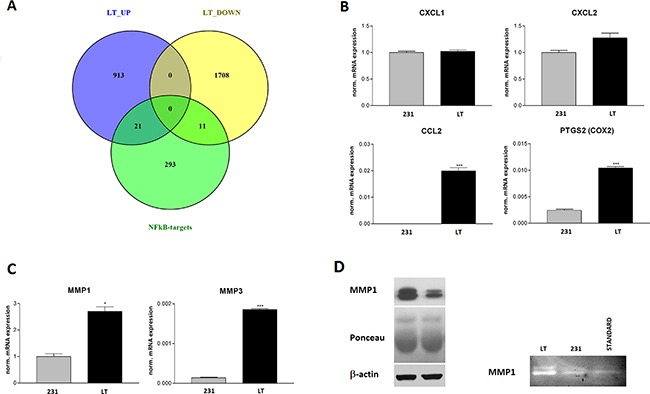
NFkB target validation **A.** Venn diagram of genes up- and down-regulated in LT versus 231 cells showing the overlap with experimentally validated NFkB target genes. **B.** Semi-quantitative real time PCR validation of the expression of the chemokines CXCL1 and -2, CCL2 and of COX2 (PTGS2) that are regulated by NFkB. **C.** Semi-quantitative real time PCR validation of MMP1 and MMP3 expression. **D.** Validation of MMP1 expression by Western blotting (loading controls: Poinceau red staining of the blot and β-actin Western blot). **E.** Zymography of serum free cell supernatants on gelatin containing gels. Standard = purified MMP1.

In fact, 32 experimentally validated targets of NFκB, among which the cyclooxygenase 2 (COX2, PTGS2), are upregulated in LT cells as compared to 231 cells indicating a potential role of NFκB in the invasive phenotype (Figure 8a). We therefore validated the expression of two inflammatory chemokines, CCL2 and CXCL2, whose expression is known to be regulated by NFkB by semiquantitative PCR analysis. CCL2 is undetectable in 231 cells and strongly expressed in LT cells. CXCL2 is also induced but did not reach statistical significance (Figure 8b). CXCL1, consistent with the microarray data, is not affected. The matrix-metalloproteases MMP1 and MMP3 are also controlled by NFkB. Matrix metalloproteases are mechanistically linked to invasion since they digest components of basal membrane and extracellular matrix. MMP1 has been described to be upregulated in brain metastases [34]. MMP1 and MMP3 were significantly upregulated at the mRNA level (Figure 8c). We also observed a significant upregulation of MMP1 at the protein level (Figure 8d) that lead to increased proteolytic activity as analyzed by zymography (Figure 8e).

**Figure 9:**
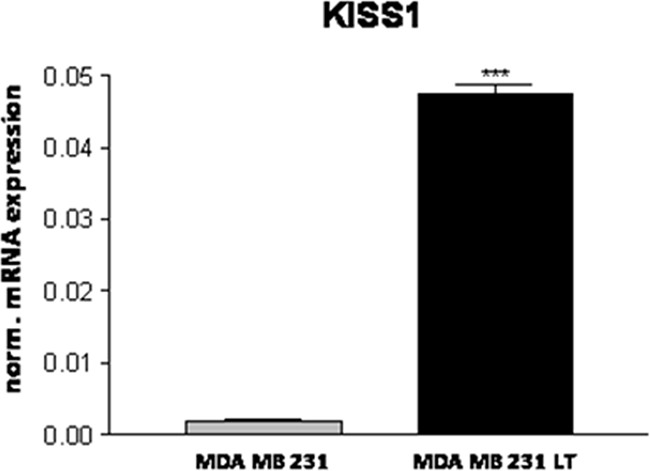
Expression of the metastasis suppressor KISS1

We also confirmed the differential expression of the metastasis suppressor gene KISS1 [35] that was barely detectable in 231 cells and strongly induced in LT cells (Figure 9).

### Tumorigenicity

**Figure 10:**
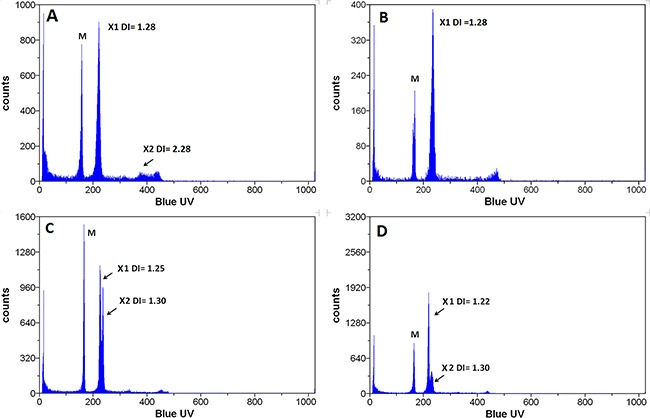
Cell populations ex vivo Flow cytometric analysis of the DNA content of cells isolated from tumors formed in nude mice after injection of LT cells. The cell population with a high DI index (AN2) was drastically reduced in the tumors grown in vivo as compared to the population injected indicating that this population is less tumorigenic.

The LT subpopulation is composed of two distinct populations, AN1 and AN2 with different grades of ploidy (see above, Figure 4). AN1 corresponds to the parental cells and AN2 is enriched in invasive and long term cells. We wished to know whether AN1 and AN2 show similar tumorigenicity *in vivo*. We took advantage of the fact that the LT cell population is composed of AN1 and AN2 populations to roughly the same extent. We injected LT cells into the mammary fat pad of immune deficient mice and isolated the tumors formed when they reached 10mm in diameter. The analysis of the DNA content of the *ex-vivo* tumors shows the presence of a single human population in addition to the population of normal murine stroma. The human populations isolated from 27 mice (Figure 10) have a mean DNA index of 1.27 (range 1.22 – 1.3; median 1.28), similar to that observed for MDA-MB-231 parental cells (Figure 4a). We observed a single mouse carrying a xenograft tumor that contained, in addition the AN1 population, a population with a DNA index of 2.28 close to the one observed for the AN2 population of LT cells grown *in vitro.* The population amounts to only 8,9%. Several mice show populations with a DNA index lower than 1.28 in addition to AN1 (Supplementary Table S3). These data show that the near tetraploid population AN2 that accounts for 59% of the LT cells does only occasionally yield a xenograft in nude mice and therefore appears of much reduced tumorigenicity as compared to the AN1 population (Supplementary Table S3).

The difference observed for the DNA index between AN1 cells and the parental cells (1.28 and 1.31, respectively) is within the coefficient of variation of the technical measure. In order to rule out that the AN1 population growing in xenografts is different from the parental 231 cells we performed aCGH analyses also for two xenograft tumors. One of the two had a single population with a DNA index of 1.28 and the other one had two subpopulations with DNA indexes of 1.22 and 1.3. Both carry the characteristic 5p14.1-15.33 amplification indicating that the tumors derived from the AN1 population present in the LT line that is identical to 231 parental cells.

We have analyzed local recurrences, lymph node metastases and distant metastases for several animals. Almost all metastases analyzed showed DNA indexes within the coefficient of variation of the index of the primary tumor. In one animal that carried a primary tumor containing populations of 0.88 and 1.28, the metastases showed both populations in three metastases and one local recurrence. One metastasis contained an additional population with a DNA index of 1.19 (Supplementary Table S3).

## DISCUSSION

There is an essential consensus concerning the hallmarks of metastatic cancer [16] but there is much less agreement on how and when during carcinogenesis these hallmarks are developed. The multistep carcinogenesis model [1] postulates the sequential acquisition of these hallmarks during a molecular evolution. The timing of this process has recently been revised at least for colon cancer where a “big bang” appears to create a dramatic genetic instability followed by an outgrowth of various re-stabilized clones in parallel [36, 37]. Acquisition of additional molecular alterations during dissemination is well documented [38] but the precise path from the primary tumor that grows *in situ* to invasive and eventually metastatic cancer is still far from being fully understood. In order to better define the molecular alterations associated with invasion, the first step in metastasization, we isolated highly invasive subpopulations of the well described triple negative breast cancer cell line, MDA-MB-231, and report here the molecular characterization of these subpopulations. Okuyama and colleagues have isolated a similar subpopulation from MDA-MB-231 cells by selection of cells that invaded Matrigel [39], however, the authors only partially characterized these cells. The group of Massagué isolated subpopulations of MDA-MB-231 cells that, when xenografted into immunodeficient mice, formed metastases with a particular tropism for lung [27], bone [28, 29] and brain [30]. These studies show that organ specific metastasis depends on specific genetic programs that can be activated in the same tumor cell [40]. Our analysis focused on the first step of metastasization where the transformed tumor cell must acquire the potential to invade the surrounding tissue after degradation of the extracellular matrix.

The preparative invasion assay is performed in medium without serum and cells are allowed to invade for 24hrs. Once the cells have migrated to the lower compartment they are exposed to serum containing attractive medium in which the observed proliferation difference could determine a more rapid growth of INV and LT cells. When normalizing for the different proliferation rate it becomes evident that 24hrs are not sufficient to yield the effect observed.

Microarray based genotyping excluded contami-nation with an unrelated cell line. Array CGH analyses identified the invasive population as an independent clone from the same patient since it does not carry the amplification 5p14.1-15.33. Loss of a specific amplified region is unlikely and therefore we exclude that this population has been generated during cell culture. MDA-MB-231 cells have originally been isolated from a pleural effusion of a breast cancer patient [38] and the protocol followed for isolation did not comprise any selection or cloning step. A heterogeneous population is therefore absolutely possible. Karyotyping showed 65-69 chromosomes [38] consistent with the DNA content of 1.31 measured for the certified cell line used here. The invasive subpopulation shows an almost doubled DNA content of 2.28. In the parental cell line, this population constitutes a minor subpopulation that might easily be overseen when karyotyping or be taken for occasional cells with a duplicated set of chromosomes often observed in cancer cell lines. We therefore assume that the subpopulation isolated here is part of a heterogeneous tumor. The fact that the fast growing invasive population has not overgrown the parental population indicates that the latter must produce factors that limit the growth of the former. Our selection using preparative Matrigel invasion assays yielded enrichment in invasive cells and long term culture of this line increased the enrichment as expected on the ground of the growth characteristics. With an increased proportion of these cells the growth limiting effects of the parental population are apparently overcome. In fact, LT cells where approximately half of the cells belong to the invasive population, grow even faster.

It is not clear whether and eventually how the increased DNA content of the invasive subpopulation is causally related to the invasive phenotype. Near tetraploid cells are considered to constitute a reservoir of cells able to originate aneuploid subclones with random losses of genetic material thus contributing to tumor heterogeneity [41]. In human patients, these cells might therefore give rise to aggressively growing and eventually metastatic subclones whereas the establishment of a cell line corresponds to a snapshot during tumor evolution and interrupts clonal selection.

Gene expression analyses showed a drastically altered gene expression profile characterized by the over-expression of matrix-metalloproteases, as expected for an invasive population, and by several genes that are *bona fide* targets of the transcription factor NFkB, among which the inflammatory cytokine CCL2 that is absent from 231 cells and highly expressed by LT cells. CCL2 has been described to indirectly promote breast cancer metastasis through the induction of pro-metastatic macrophages [42, 43] yet there is evidence for anti-metastatic action of CCL2 that are mediated by the entrainment of neutrophils that reduce metastatic seeding of the lung [44].

Interestingly, the list of differentially expressed genes shows a significant overlap with genes that have been identified as apocrine breast cancer specific genes [32]. Apocrine tumors constitute a subset of triple negative breast cancers [45]. The androgen receptor as well as other markers of apocrine breast cancer is expressed by MDA-MB-231 cells but not overexpressed by the invasive subpopulation. Hence, LT cells share some but not all of the features of apocrine breast cancer. Apocrine metaplasia has been described as a potential precursor lesion of apocrine triple negative breast cancer [46]. One can therefore speculate that LT cells might have been derived from a co-occurring apocrine tumor with reduced tumorigenicity but enhanced proliferation.

When tested for stem cell markers CD44 and CD24 [47], the LT population as well as the parental cell line showed more than 90% of CD44^+^/CD24^−^ cells consistent with published data [48] with only minor differences (Supplementary Figure S2). CD44 and CD24 gene expression data show a significant lower expression of CD24 in LT cells that is, however, not reflected on the protein level as measured by flow cytometry (Supplementary Figure S2). Hence, the reduced tumorigenicity cannot be attributed to a reduced number of cells with stem cell features. The reduced tumorigenicity that also determined the lack of a metastasis forming potential indicates that the single steps in tumor progression are not necessarily acquired in a linear manner. The fast growing, highly invasive subpopulation is unable to progress to metastasis, at least in part due to the fact that it strongly expresses the metastasis suppressor KISS1 [49]. However, KISS1 does not affect tumorigenicity [50]. Therefore KISS1 alone cannot be responsible for the particular behavior of this clone.

The invasive subpopulation shows different drug sensitivities. Most likely due to the higher DNA content and chromosome number, the cells are more sensible to drugs that affect the mitotic apparatus.

The greatly reduced tumorigenicity of the invasive subpopulation is unexpected and is a central information delivered by our analysis: invasion is not necessarily linked to a more aggressive tumor growth and metastasis. Here we describe two subpopulations of a breast cancer of the same patient with differential invasion potential and inversely differential tumorigenicity as a proof of the concept that invasion is necessary but not sufficient for metastasis. This analysis shows that heterogeneous tumors can generate clones with different invasive and metastatic potential, two independent processes of tumor progression. Future studies will exploit LT and 231 cells to study molecular features related to the strikingly different tumorigenicity of two clones from the same patient.

## MATERIALS AND METHODS

### Cell lines

Human metastatic breast cancer cells MDA-MB-231 (14), gently provided from Interlab Cell Line Collection (ICLC, www.iclc.it, Genoa, Italy [51]), were cultured in DMEM (Gibco-BRL, Rockville, MD, USA) supplemented with 10% fetal bovine serum (FBS), 2 mM L-glutamine and 100 U/ml penicillin/streptomycin at 37°(all from Gibco-BRL). The same culture conditions were used for the MDA-MB-231 derivative cells: 231 INV and 231 LT. INV and LT cells were deposited at ICLC.

### Invasion assay

BD Bio Coat invasion chambers (BD Biosciences Milan, Italy) coated with growth factor reduced *Matrigel* were used to isolate the invasive subpopulation from the MDA-MB-231 cells and for analytical invasion assays. The assay was performed according to the recommendations of the manufacturer. Briefly, cells were cultured at a density of 1×10^6^/ml in medium containing 0,1% FCS for 18 h before the assay, one 100,000 cells suspended in 0.5 ml of medium containing 0.1% FCS were added to the top chambers of 24-well trans-well plates (BD Biosciences; 8μm pore size) and the lower chambers were filled with 10% FCS or 50% NHI3T3 supernatant in medium that served as a chemo-attractant. After 24 hours incubation, top (non-migrated) cells were removed, and bottom (migrated) cells, collected and disseminated on culture dishes. The passage of the cells through *Matrigel* was repeated twice in order to enrich the invasive subpopulation. After the third passage, cells were collected and cultured in standard conditions. For analytical invasion assays, after 24 hours incubation, top (non-migrated) cells were removed, and bottom (migrated) cells were fixed and stained with 1% toluidine blue to visualize nuclei. Migrated cells were counted under ×200 magnification in five fields, and the mean for each chamber was determined. Experiments were run in triplicate and results were expressed as mean ± SEM of three independent experiments.

### Proliferation assay

Proliferation was assessed using the colorimetric crystal violet assay (Sigma Aldrich, Milano). Briefly 2500 cells in 0.1 ml growth medium were seeded into 96-well plates in octuplicate. Cells were incubated at 37°C and 5%CO_2_ for 5 days and the test was performed each 24hrs. The cells were washed with PBS (pH 7.3) and subsequently fixed and stained for 20min in a solution of 0.75% crystal violet, 0.35% sodium chloride, 32% ethanol, and 3.2% formaldehyde (Sigma-Aldrich, Milano). The stain was then dissolved in 50% ethanol, 0.1% acetic acid and read with a microtiter plate reader at 595 nm [52]. The proliferation rate was also assessed in parallel in real time mode using the Xcelligence Sytem (ACEA Biosciences, SanDiego,CA, USA) [53]. Briefly, 5000 cells were seeded on an E-plate 96, in triplicate and monitored continuously for overall impedance profile over 5 days.

### Apoptosis and necrosis assay

Flow cytometry analysis of apoptosis and necrosis was performed using the cytofluorimetric assay Annexin-V-FLUOS Staining Kit (Roche, Germany) following the instructions given by the provider. For the induction of apoptosis, cell lines were treated with 250μM or 500μM H_2_O_2_ for 4h [54].

### Chemosensitivity assay

Doxorubicin, cisplatin and 5-fluorouracil were purchased from Sigma-Aldrich (St. Louis, MO, USA). Drugs were dissolved in normal saline (doxorubicin and cisplatin) or methanol and normal saline (5-fluorouracil). Taxol, mitoxantrone, vincristine, irinotecan, and ifosfamide were obtained in clinical form. Taxol was diluted in normal saline containing 1% cremophor/ethanol (1:1, v/v), mitoxantrone and vincristine were diluted in normal saline while irinotecan and ifosfamide were diluted in distilled water. All drug solutions were prepared freshly just before use.

MDA-MB-231, and 231 LT cell lines were plated into 96-well flat-bottomed microtiter plates for 6-8 hours. In order to reach the final concentrations indicated in the results section, the anticancer drugs were then added to each well diluted in 20 μl. After 3 days the cells, treated in duplicate, and finally suspended in 200 μl/well medium, were added with 50 μl of 3-(4,5-dimethylthiazol-2-yl)-2,5-diphenyltetrazdium bromide (MTT, Sigma, St. Louis, MO, USA) solution (2 mg/ml in PBS) and incubated for further 4 hrs at 37°C. After centrifugation at 275xg for 2 minutes, the medium was aspirated and replaced with 100 μl of 100% dimethylsulfoxide. Complete solubilization of formazan crystals was achieved by shaking after 30 minutes of incubation at room temperature. The absorbance was measured on a plate reader 400 ATC (SLT Labinstruments, Austria) at 540nM. IC_50_ values were calculated on the basis of the analysis of single concentration-response curves. Experiments were repeated 4-12 times to allow the calculation of the mean IC50.

Student's t test was used for the statistical analysis of data. The resistance index (RI) for each drug was defined as the ratio between the IC_50_ values of the subpopulation tested in comparison to parental cells. Pharmacologically significant resistance was arbitrarily defined when the RI was ≥ 2.5, while significantly increased sensitivity was defined when the RI was ≤ 0.80.

### Sample processing for DNA FCM, aneuploidy determination and cell cycle analyses

Tissue fragments were minced on Petri dishes using scalpels, collected in 2ml detergent solution (0.1 M citric acid, 0.5% Tween-20) and then submitted to mechanical disaggregation in a gentle MACS dissociator as reported [55]**.** Nuclei suspensions were obtained and filtered over a 50μm nylon sieve (CellTrics, Partec GmbH, Muenster, Germany). An absolute count of the nuclei was performed by FCM (CyFlow® ML, Partec GmbH) after 1–10 dilution in water. The final volume was calculated to obtain the concentration of 600,000 nuclei/ml. One volume of detergent solution was first added followed by 10 min incubation and gentle shaking. Finally, 6 volumes of staining solution (0.4 M Na2HPO4, 5 μM DAPI in water) were added. Each sample was then analyzed, after 15 min of incubation using a CyflowML multiparameter flow cytometer (Partec). The DNA aneuploid subpopulations (DI ≠ 1) were sorted using a Cyflow Space FCM equipped with a PPCS unit (Partec GmbH) at the purity of about 99%. Excitation of DAPI was provided by an UV mercury lamp (HBO-100 long life, 100W) and the emitted fluorescence was collected by the Gratz setting (488 blue solid laser shut down; 435 nm long pass filter). Human lymphocytes from female healthy donors were used as diploid DNA controls. Only samples with at least 2 separate G0–G1 peaks, after mixing with the controls DNA, were considered aneuploid. DNA Index (DI) values were evaluated as the ratio of the mean channel number of the human DNA aneuploidy G0–G1 peak to the mean channel number of the human diploid G0–G1 control peak. Murine cells in xenograft samples were easily discriminated on the base of the DNA content, lower than that of human lymphocytes. For each cell population, analysis of the percentage of cells in the different cell cycle phases was determined by the ModFit LT™.

### Single nucleotide variant and copy number variation analysis

Genomic DNA was extracted from each cell line using QIAamp DNA Mini kit (Qiagen). Processing of genomic DNA was performed on Affymetrix platform 450 using the GeneChip Mapping 250K Assay Kit (Affymetrix, Santa Clara, CA, USA) following the protocol provided. Briefly, 250ng of DNA sample were digested with NspI restriction enzyme and adapters were ligated using T4 DNA Ligase. A primer set, that recognizes the adaptor sequence was used to amplify adaptor-ligated DNA fragments by polymerase chain reaction (PCR) on a BioRad MyCycler thermocycler. 90μg of amplified and normalized PCR product was fragmented and labeled. Hybridization, washing, staining and scanning of single-nucleotide polymorphism (SNP) arrays were performed on the Affymetrix station. Quality of the samples was assessed on agarose gels before the hybridization step. Affymetrix Genotyping Console (GTC4.1.2) was used to perform genotype call and quality control assessments. Copy number analyses and virtual karyotypes were generated using CNAG3.0 [56].

### Gene expression profiling

Total RNA was isolated from 231 and LT cells using the RNeasy Mini Kit (Qiagen) according to the manufacturer's instructions. cDNA synthesis was performed using T7-(dT)24 oligo primers and the 3′ IVT plus Kit (Affymetrix Thermo Fisher Scientific, Santa Clara, CA, USA) according to the manufacturer's instructions. Double stranded labeled cDNAs were purified with Purification Beads followed by three 80% ethanol washes, and then fragmented using 3′ IVT Amplification Kit (Affymetrix Thermo Fisher Scientific, Santa Clara, CA, USA). cDNA synthesis, cRNA retrotranscription, labeling, purification and fragmentation was performed according to the manufacturer's instructions (Affymetrix). Fragmented, labeled cRNA was used for screenings of GeneChip Human Genome U133A arrays (Affymetrix). The experiment consisted of 3 biological replicates. Hybridization and scanning was performed on the Affymetrix platform. Data were normalized following RMA algorithm [57] implemented in R/Bioconductor [58]. Statistically significant expression changes were determined using permutation tests (SAM) [59]. Genes regulated at least two fold in comparison to untreated controls were considered. The delta value was set to return a median false significant number of zero. Hierarchical clustering was performed using Pearson correlation as distance measure and average linkage. Annotations were obtained through the DAVID database [60]. Gene enrichment analysis was performed using the EnrichR online tool [31]. Gene lists were compared using Venn diagrams [61].

Validation of gene expression data by semi-quantitative real time PCR using SYBR-Green was performed as described previously [62] using the primers reported in Supplementary Table S4.

### Western blots and zymography

Conditioned media from 231 and LT cells were analyzed by Western blotting using antibodies against MMP-1 (kind gift from Ralf Lichtinghagen, Medical School, Hannover) as previously described [62, 63]. Gelatin zymography was performed as previously described [64]. Purified MMP-1 standard was purchased at Calbiochem.

### In vivo-experiments

Swiss nu/nu immunocompromised mice were purchased from Charles River (Calco, Como) and maintained in 12-hour dark/light cycles with water and food ad-libitum. Animals were housed and maintained in the Animal Care Facility of the IRCCS San Martino-IST, accordingly to national and European regulations (D.L. 4/3/14 No. 26; 86/609/EEC Directive). All animal experiments were approved by the internal Ethic Committee and by the Italian Ministry of Health.

A group of 27 six-week-old female mice were anesthetized with a mixture of Ketamine-Xylazine given intraperitoneally, the mammary fat pad of the inguinal fourth gland was exposed and 500.000 LT cells were injected in 10μl of PBS using a disposable syringe with a 29G needle. Animals were monitored daily and euthanized when tumors reached the size of 1200mm^3^ and before any sign of suffering became detectable. Tumors were removed and either frozen for genomic analysis or directly processed for isolation of nuclei. The site of injection was palpated three times/week and tumor size was recorded using a caliper.

## SUPPLEMENTARY MATERIALS FIGURES AND TABLES










