# Supplementary material for: A highly invasive subpopulation of MDA-MB-231 breast cancer cells shows accelerated growth, differential chemoresistance, features of apocrine tumors and reduced tumorigenicity in vivo
Source: Oncotarget. 2016 Sep 10;7(42):68803–20. doi: 10.18632/oncotarget.11931 (PMC5356591; doi:10.18632/oncotarget.11931)
Supplement: Supplementary file 1 [file oncotarget-07-68803-s001.pdf]

A highly invasive subpopulation of MDA-MB-231 breast cancer cells shows accelerated growth, differential chemoresistance, features of apocrine tumors and reduced tumorigenicity *in vivo*

SUPPLEMENTARY FIGURES AND TABLES

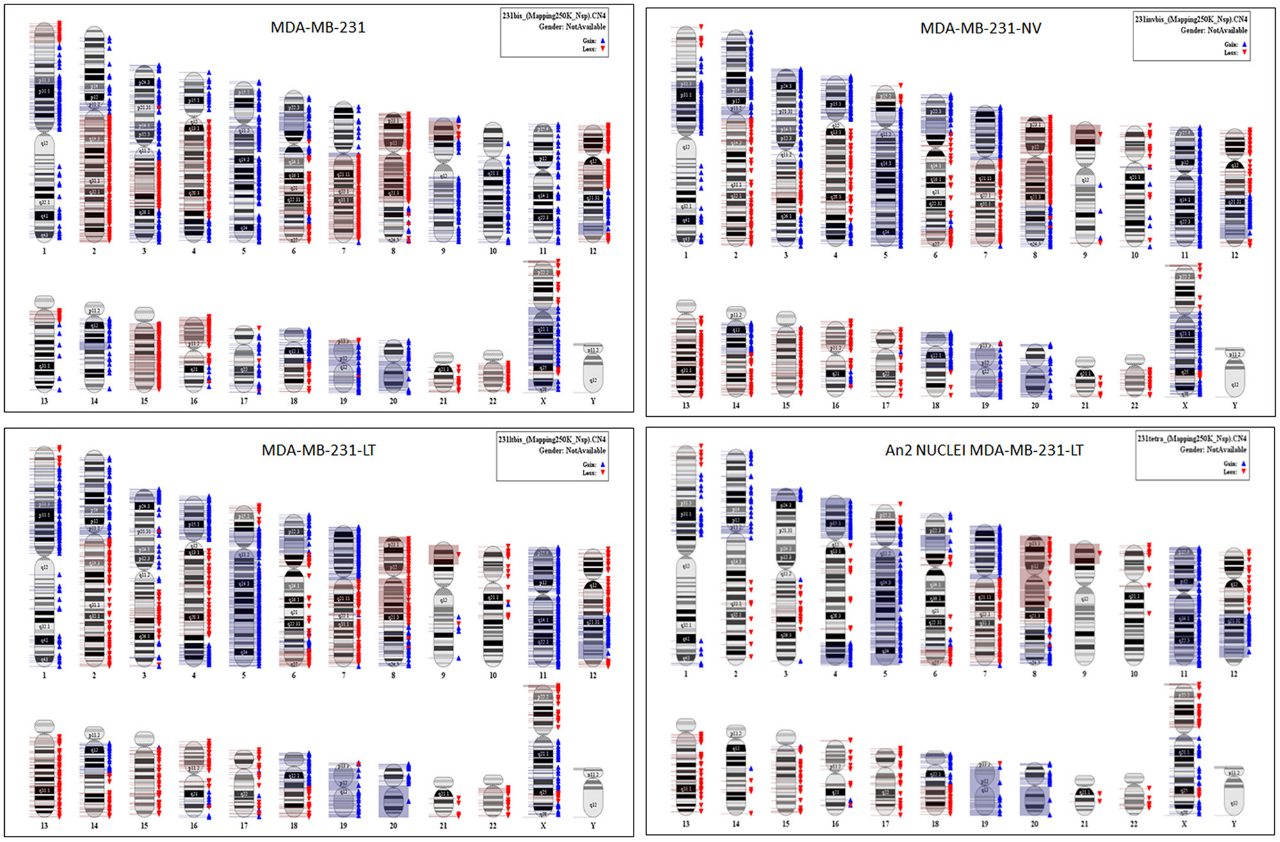

Supplementary Figure S1: Virtual karyotypes of 231, INV, LT cells and nuclei purified from LT cells.

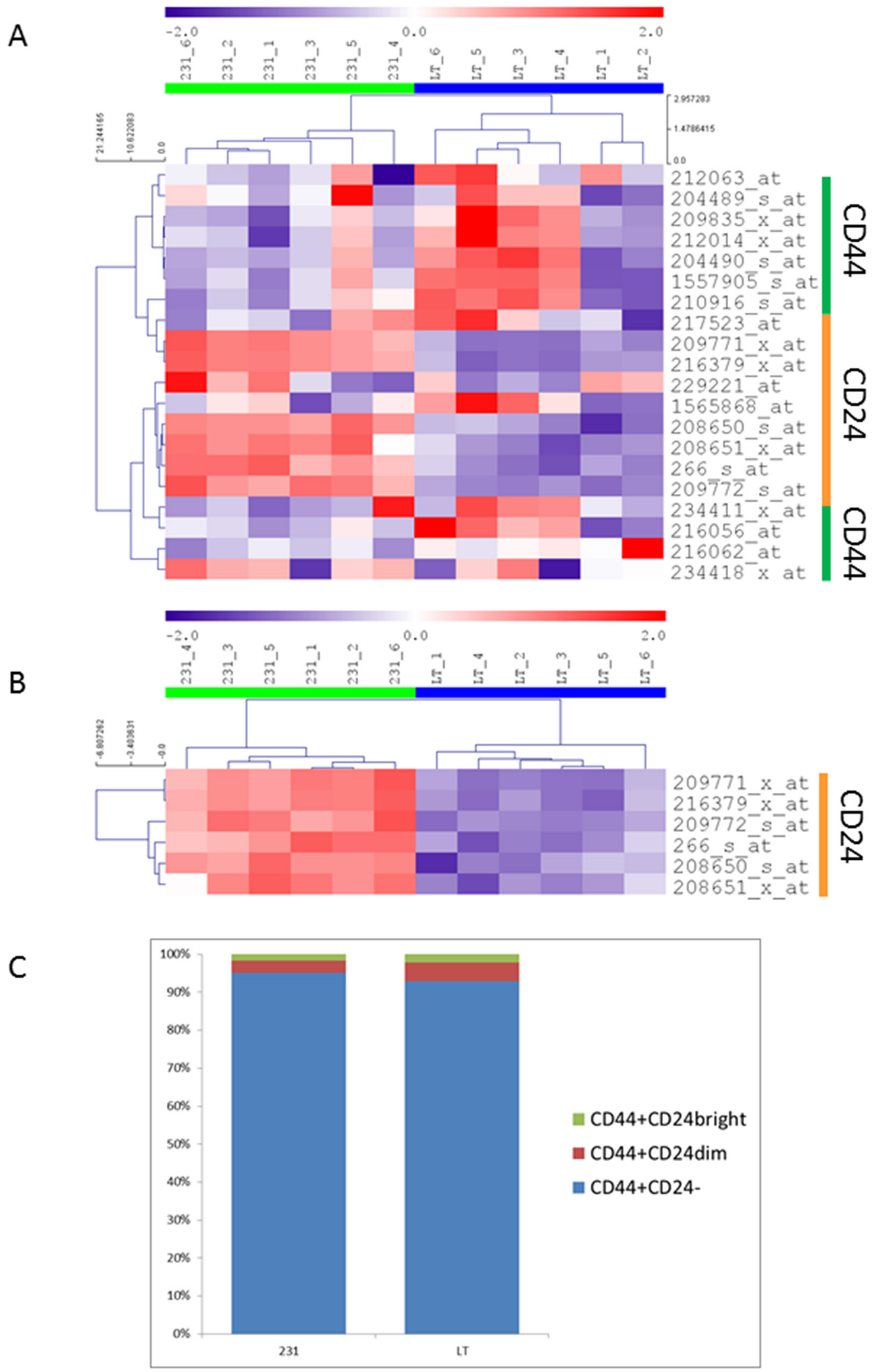

Supplementary Figure S2: Stem cell characteristics. A. Hierarchical clustering using the expression values of probesets specific for CD44 and CD24. **B.** Significance analysis of microarray analysis using the expression values of probesets specific for CD44 and CD24. **C.** Flow cytometry using anti-CD44 and anti-CD24 antibodies.

Supplementary Table S1: Genes on chromosome 5p14.1-15.33.

See Supplementary File 1

Supplementary Table S2: Results of Significance Analysis of Microarrays.

See Supplementary File 2

Supplementary Table S3: DNA content of mouse xenografts of LT cells.

See Supplementary File 3

Supplementary Table S4: Reverse transcription PCR primers.

See Supplementary File 4
